# Myofunctional Therapy for the Treatment of Obstructive Sleep Apnea: Systematic Review and Meta-Analysis

**DOI:** 10.1055/s-0044-1801780

**Published:** 2025-02-04

**Authors:** Lucas Gabriel dos Anjos Ferreira, Vanessa Souza Gigoski de Miranda, Maria Eduarda Pedroso Baseggio, Miriam Allein Zago Marcolino, Deisi Cristina Gollo Marques Vidor

**Affiliations:** 1 Department of Pediatrics, Universidade Federal de Ciências da Saúde de Porto Alegre (UFCSPA), Porto Alegre, RS, Brazil; 2 Department of Speech Therapy, Universidade Federal de Ciências da Saúde de Porto Alegre (UFCSPA), Porto Alegre, RS, Brazil; 3 Department of Epidemiology, Universidade Federal de Ciências da Saúde de Porto Alegre (UFCSPA), Porto Alegre, RS, Brazil; 4 National Institute of Science and Technology for Health Technology Assessment - INCT/IATS, Porto Alegre, RS, Brazil

**Keywords:** myofunctional therapy, sleep apnea, obstructive sleep apnea, syndromes, exercise therapy

## Abstract

**Introduction** Given the severity with which obstructive sleep apnea (OSA) syndrome can affect the patient's health, many therapies have been presented to minimize the occurrence of episodes of airway obstruction during sleep. Regarding non-invasive and effective methods, orofacial myofunctional therapy (OMT) is an important adjuvant in the clinical treatment.

**Objective** To verify the effectiveness of OMT in the treatment of adult patients affected by OSA.

**Data Synthesis** A search strategy was developed with terms adapted to the requirements of the main databases in the health field (PubMed, Cochrane, Embase and secondary databases) to designate the adult OSA population and the OMT intervention. The analysis of the records found was performed by two independent examiners and, at the end, we included five randomized clinical trials that presented the outcome of effectiveness of the OMT verified through the apnea-hypopnea index (AHI).

**Conclusion** The effectiveness of the OMT in the treatment of adult OSA patients was verified, both alone and in association with other interventions, through the reduction in the AHI and the Epworth Sleepiness Scale.

## Introduction

Human beings spend about a third of their lives sleeping. 1 Sleep is not only a period of rest that is essential to maintain several aspects related to the individual's quality of life, 2 but it is also the moment in which physiological and metabolic changes responsible for the proper functioning of the human body are consolidated. 3 4 Due to its importance, any change in sleep can cause damage to various aspects of the patient's life, in different degrees, directly or indirectly. 1 2 5

Among the disorders that can impact sleep, one of the most prevalent is obstructive sleep apnea (OSA), which affects 9% to 38% of the adult population, constituting a public health problem. 6 7 8 The condition is characterized by partial or total obstruction of the upper airway during sleep, 4 5 8 9 10 resulting in greater respiratory effort, inadequate ventilation, reduced oxygen saturation in the blood (SPO _2_ ), acute disturbances in gas exchange, and fragmentation sleep, with the occurrence of nocturnal awakenings. 4 9 Factors such as age (> 65 years), gender (male), and obesity increase the risk of developing OSA. 10

The diagnosis of this condition is established through the polysomnography (PSG) exam, which determines the apnea-hypopnea index (AHI), and patients with OSA present an AHI ≥ 5 events/hour (from 5 to 14–mild; from 15 to 29–moderate; > 30–severe). 11 This situation can cause or aggravate cardiovascular abnormalities such as refractory heart failure, resistant arterial hypertension, nocturnal angina, and nocturnal arrhythmias. 12 Due to these complications, OSA is related to considerable morbidity and mortality rates. 10 13

Given the severity with which OSA can affect the patient's health, many therapies have been presented to minimize the occurrence of episodes of airway obstruction during sleep and reduce the damage caused by the condition. 4 The reference standard currently used for the treatment is the continuous positive airway pressure (CPAP) device, which adapts to a flexible tube through which the released air is conducted to a mask firmly fitted to the patient's nose, exerting continuous positive pressure on the patient's airways. The biggest obstacle to its use, despite its already established efficiency, is treatment adherence. 14 15 In addition, its action is momentary, that is, it only works while the individual is using it, and it does not provide permanent or gradual improvement of the condition. Other measures, such as performing surgeries and adopting behaviors that can minimize risks (such as weight loss, physical activity etc.), are invasive and/or demand persistence and motivation with long-term results, in addition to not being effective in many cases. 14

Regarding non-invasive and effective methods to treat OSA, orofacial myofunctional therapy (OMT) is an important adjuvant in the treatment, having achieved good results when used. 16 The OMT applied to these cases is based on the fact that airway collapse during sleep is often associated with flaccidity generated by the pathology in the oropharyngeal muscles. 16 17 Thus, the OMT seeks to modify the patterns of posture and strength of the oropharyngeal and velopharyngeal muscles through the performance of specific isotonic and isometric exercises, beyond training the stomatognathic functions, with to the goal of maintaining upper airway permeability during sleep. 17

Based on these assumptions, the aim of the present study is to verify the effectiveness of OMT in the treatment of adult OSA patients through a systematic literature review, answering the guiding question: “Is OMT effective as a treatment for OSA in adults?”.

## Review of the Literature

The present systematic review was conducted in accordance with the instructions of the Cochrane Collaboration 18 and was reported according to the Preferred Reporting Items for Systematic Reviews and Meta-Analyses (PRISMA) statement. 19 The study protocol was registered at the International Prospective Register of Systematic Reviews (PROSPERO; http://www.crd.york.ac.uk/PROSPERO/ ), under approval number CRD42020159132.

Searches including studies indexed until December 2020 were performed in the following electronic databases: MEDLINE (accessed via PubMed), EMBASE, The Cochrane Central Register of Controlled Trials (Cochrane CENTRAL), the Latin American and Caribbean Literature in Health Sciences (LILACS), Healthy Cities (CidSaúde), the Pan American Health Organization (PAHO), the Pan American Information and Documentation Network on Sanitary Engineering and Environmental Sciences (REPIDISCA), the Nursing Database (BDENF), the Caribbean Health Sciences Literature (MedCarib), the World Health Organization Library Information System (WHOLIS), the Spanish Bibliographic Index on Health Sciences (IBECS) and the Scientific Electronic Library Online (SciELO). Bibliographic references of the included studies, as well as those extracted from Google Scholar and other bibliographic resources in the health filed related to OMT for the treatment of sleep disorders were used as a source additional data to minimize selection bias. The search strategy was developed using keywords identified in the Medical Subject Headings (MeSH), Health Science Descriptors (DeCS), and EMBASE Subject Headings (EMTREE) related to the population of interest, intervention and outcomes. To increase the sensitivity of the search, entertaining terms and synonyms were incorporated into the search strategy, which was adapted to the requirements of each database. The complete search strategy, with the terms used in the search in MEDLINE via PubMed, can be seen in Table 1 .

**Table 1:** Search strategy used in the MEDLINE database accessed via PubMed

**(#1)** **Population**	(“adult”[Mesh] OR “adults” OR “aged”[Mesh] OR “Elderly” OR “aged, 80 and over”[Mesh] OR “Oldest Old” OR “Nonagenarians” OR “Nonagenarian” OR “Octogenarians” OR “Octogenarian” OR “Centenarians” OR “Centenarian”) AND (“Sleep Apnea, Obstructive”[Mesh” OR “Apneas, Obstructive Sleep” OR “Obstructive Sleep Apneas” OR “Sleep Apneas, Obstructive” OR “Obstructive Sleep Apnea Syndrome” OR “Obstructive Sleep Apnea” OR “OSAHS” OR “Syndrome, Sleep Apnea, Obstructive” OR “Sleep Apnea Syndrome, Obstructive” OR “Apnea, Obstructive Sleep” OR “Sleep Apnea Hypopnea Syndrome” OR “Syndrome, Obstructive Sleep Apnea” OR “Upper Airway Resistance Sleep Apnea Syndrome” OR “Syndrome, Upper Airway Resistance, Sleep Apnea” OR “Sleep Apnea Syndromes”[Mesh] OR “Apnea Syndrome, Sleep” OR “Apnea Syndromes, Sleep” OR “Sleep Apnea Syndrome” OR “Sleep Hypopnea” OR “Hypopnea, Sleep” OR “Hypopneas, Sleep” OR “Sleep Hypopneas” OR “Apnea, Sleep” OR “Apneas, Sleep” OR “Sleep Apnea” OR “Sleep Apneas” OR “Sleep Apnea, Mixed Central and Obstructive” OR “Mixed Central and Obstructive Sleep Apnea” OR “Sleep Apnea, Mixed” OR “Mixed Sleep Apnea” OR “Mixed Sleep Apneas” OR “Sleep Apneas, Mixed” OR “Hypersomnia with Periodic Respiration” OR “Sleep-Disordered Breathing” OR “Breathing, Sleep-Disordered” OR “Sleep Disordered Breathing” OR “apnea” OR “apneas”)
**(#2) Intervention**	“Myofunctional Therapy”[Mesh] OR “Myofunctional Therapies” OR “Therapies, Myofunctional” OR “Therapy, Myofunctional” OR “Orofacial Myotherapy” OR “Myotherapies, Orofacial” OR “Myotherapy, Orofacial” OR “Orofacial Myotherapies” OR “Oral Myotherapy” OR “Myotherapies, Oral” OR “Myotherapy, Oral” OR “Oral Myotherapies” OR “Orofacial Myology” OR “Myologies, Orofacial” OR “Myology, Orofacial” OR “Orofacial Myologies.”
**Search**	***#1 AND #2***

Considering the eligibility criteria, the studies were initially analyzed through title and abstract by two independent evaluators, who classified the studies as “eligible,” “excluded” or “uncertain.” Discrepancies were discussed among reviewers. The full texts of the studies considered eligible or uncertain at this stage were obtained and independently assessed by the two reviewers. The reasons for the exclusion of the full texts evaluated were recorded. Again, disagreements arising from the comparison between the two lists of the independent reviewers were discussed to reach a consensus. In both stages, when consensus was not reached, a third independent evaluator was recruited for deliberation.

Only randomized clinical trials were included. No language or publication date restrictions were applied. The study population consisted of adults of both sexes with OSA. In the present study, the intervention considered was OMT. Studies with a comparison group should present the OMT intervention in at least one of the groups. In studies with other interventions, only data referring to patients exposed to OMT and the control/placebo group were considered for the review. The main outcome of the present review, to verify the effectiveness of OMT, was the difference in the frequency of OSA episodes after OMT and a comparison with the controls, assessed through objective measurements (AHI) obtained by PSG. Other outcomes evaluated were the lowest SpO _2_ value obtained during the examination and the results of the Epworth Sleepiness Scale (ESS).

The risk of bias for each study was assessed using The Cochrane Collaboration's tool for assessing risk of bias, 20 specific for intervention studies, including seven domains (random sequence generation, allocation confidentiality, blinding of participants and investigators, blinding of outcome evaluators, incomplete outcome data, selective reporting, and other biases). For each domain, the risk of bias was deemed high, low or unclear by two independent evaluators. When consensus was not reached, a third independent evaluator was recruited for deliberation.

Data extraction was conducted by a reviewer and checked by a second reviewer following a standard form in Excel (Microsoft Corp., Redmond, WA, United States). The following data were extracted: methodological design, number and characteristics of the subjects, characteristics of the intervention (number of sessions, frequency, exercise time), type of evaluation, and outcomes (pre- and posttherapy PSG data).

For the quantitative analysis, we extracted the number of subjects per group and the mean and standard deviation (SD) values of the outcomes of interest for the pre- and postintervention period for the OMT group and the control group. When necessary, median and interquartile range values were extracted and converted to mean and SD values using the method described by Wan et al. 21

Two analyzes were conducted: the first, to estimate the mean difference in outcomes after OMT, for which the cumulative effect estimate by meta-analysis was obtained by comparing the means of each outcome in the post- versus preintervention periods, only for the OMT group of the included studies; And the second, for the comparison of the means of the outcomes after the interventions between the OMT group and the controls. Both analyzes were summarized as weighted mean difference (MD) with a 95% confidence interval (95%CI) using the inverse method of variance, following the random effects model. Values of *p* = 0.05 were considered statistically significant. Statistical heterogeneity among studies was assessed using the I ^2^ inconsistency test, in which values above 25% and 50% were indicative of moderate and high heterogeneity respectively. The presence of high heterogeneity was investigated by sensitivity analysis, with individual studies being removed, considering variations in patient characteristics and methodological differences. Subgroup analyzes were conducted considering the association of OMT with CPAP.

The results of studies that did not present sufficient data for inclusion in the meta-analysis were qualitatively described. All analyzes were conducted in the RStudio software (Posit PBC, Boston, MA, United States), version 1.1.383, an integrated environment for the use of the statistical software R (R Foundation for Statistical Computing, Vienna, Austria), version 4.0.3, and all analyzes were conducted using the “meta” analysis package. 22

**Fig. 1:**
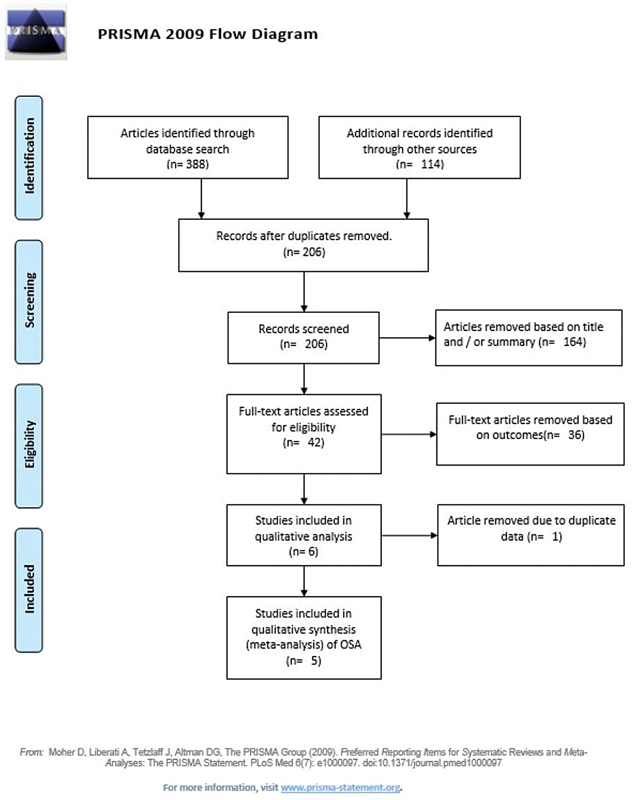
Flowchart of the selection of studies.

As shown in Fig. 1 , 502 records were found, 388 in the PubMed, Cochrane and EMBASE databases, and 114 records in other sources. After excluding 296 duplicates, the titles and abstracts of 206 records were analyzed. Of these, 42 articles were selected by the 2 evaluators for full-text reading, and 36 articles were excluded for not presenting the outcomes of interest and 1, for presenting the same data as an article already included. At the end of the selection process, five articles were included in the present review. 6 23 24 25 26 Table 2 presents a summary of the characteristics of the included studies, such as the number of patients in each of them (intervention and comparison group), gender, age, mean body mass index (BMI), type of myofunctional intervention, duration of the intervention, and weekly frequency of therapy. Table 3 shows the outcome data used in the meta-analyses: AHI, SpO2 drop and ESS score.

**Table 2:** Characteristics of included studies

		
	Author, year	N	Sex	Age	BMI	Intervention	Duration of the intervention	Frequency	N	Sex	Age	BMI	Intervention	Duration of the intervention	Frequency
	Guimarães et al., 2009 23	16	10	51.5 ± 6.8	29.6 ± 3.8	OMT	3 months	30 minute per week + daily in home.	15	73%	47.7 ± 9.8	31 ± 2.8	Deep breathing and nasal cleansing	NA	NA
	Ieto et al., 2015 24	19	11	48 ± 14	28.1 ± 2.7	Nasal lavage + OMT	3 months	8 minutes, 3 times a day	20	11	45 ± 13	28.3 ± 2.5	Nasal dilators during sleep and nasal cleansing	NA	NA
	Diaféria et al., 2017 25	27	27	45.2 ± 13	25 ± 7.4	OMT	4 months	Every day for 20 minutes	24	24	42.9 ± 10.5	28.6 ± 4	Exercises without therapeutic function	NA	NA
	Diaféria et al., 2017 25 (combination group)	22	22	47.5 ± 10.9	27.9 ± 2.4	OMT + CPAP	Average number of hours of daily CPAP use.	Every day for 20 minutes + CPAP	27	27	46.4 ± 9.1	28.7 ± 3.3	CPAP	Average number of 4 hours of daily CPAP use.	NA
	Neumannova et al., 2018 26	15	−	53.87 ± 7.79	40.3 ± 9.4	OMT + CPAP	1/5 month	4 times, 5 days in a week	20	−	54.05 ± 5.53	36.4 ± 5.4	CPAP	NA	NA
	Torres-Castro et al., 2019 6	14	5	53.80%	64.5 median	OMT + physical activity	2 months	4 times a day, 5 days in a week	13	57.10%	67	27.1	Diet, hygiene, and physical activity.	NA	NA

**Abbreviations:** BMI, body mass index; CPAP, continuous positive airway pressure; N, number; OMT, orofacial myofunctional therapy; NA, not available.

**Table 3:** Study outcomes

*Author, year*	AHI (events/hour)		Low SPO _2_ (%)		ESS score	
	Pre-OMT	Post-OMT	Pre-OMT	Post-OMT	Pre-OMT	Post-OMT
* Guimarães et al., 2009 23*	22.4 ± 4.8	13.7 ± 8.5	83 ± 6	85 ± 7	14 ± 5	8 ± 6
* Ieto et al., 2015 24*	25.4	18.1*	85.5 ± 7.5	83.8 ± 8.9	7	7
* Diaféria et al., 2017 25*	28 ± 22.7	13.9 ± 18.5	83.7 ± 7.7	84.9 ± 8.8	13.7 ± 3.2	7.5 ± 3.7
* Diaféria et al., 2017 25 (combination group) *	30.4 ± 19.8	3.4 ± 2.7	80.5 ± 11.0	89.3 ± 4.1	12.0 ± 2.6	7.3 ± 5.7
* Neumannova et al., 2018 26*	54.2 ± 27.4	4.3 ± 3.9	NA	NA	12.9 ± 4.7	5.7 ± 4.1
* Torres-Castro et al., 2019 6*	30.5*	34.5*	NA	NA	8	8

**Abbreviations:** AIH, apnea-hyponeia index; ESS, Epworth Sleepiness Scale; OMT, orofacial myofunctional therapy; NA, not available; SpO _2_ , peripheral oxygen saturation.

Note: *Data in median values.

Of the five 6 23 24 25 26 articles selected, three 23 24 25 compared groups submitted exclusively to OMT with a control group. Furthermore, two studies 25 26 associated the OMT with the use of CPAP (intervention group: CPAP + OMT; control group: only CPAP), and the another 6 used physical activity as an intervention, in addition to orofacial exercises.

**Fig. 2:**
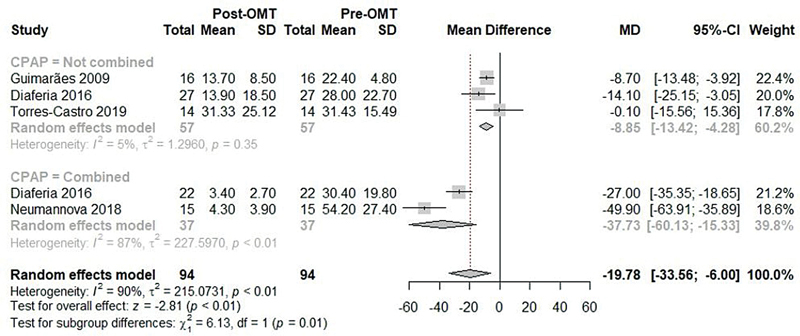
Meta-analysis of the AHI before and after OMT.

The meta-analysis of the primary outcome (mean AHI before and after OMT) included 4 studies, 6 23 25 26 and showed a significant reduction in AHI after OMT associated or not with CPAP (MD: -19.78; 95%CI: -33.56 to -6.00; I ^2^ : 90%; Fig. 2 ). In the subgroup analysis, OMT not associated with CPAP showed a significant reduction in the mean AHI, with low heterogeneity (MD: -8.85; 95%CI: -13.42 to -4.28; I ^2^ : 5%, Fig. 2 ), less expressive than the reduction presented when OMT was associated with CPAP (MD: -37.73; 95%CI: -60.13 to -15.33; I ^2^ : 87%, Fig. 2 ), with a significant difference between subgroups ( *p* = 0.01).

**Fig. 3:**
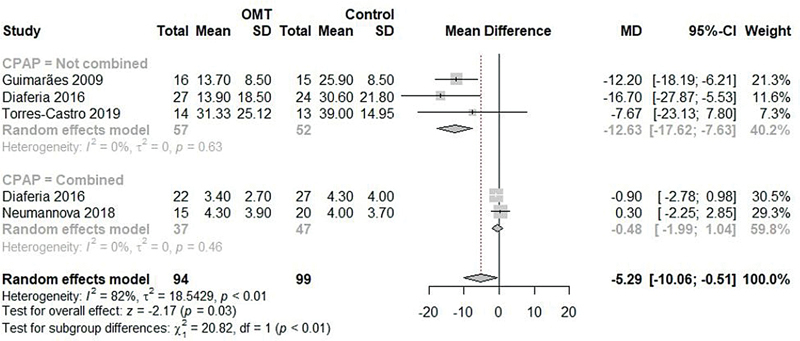
Meta-analysis of the AHI in the comparison of the OMT and control groups.

In the comparison with the control group including the same 4 studies, 6 23 25 26 a significant difference was identified in the final mean AHI, with high heterogeneity (MD: -5.29; 95%CI: -10.06 to -0.51; I ^2^ : 82%; Fig. 3 ). The subgroup analysis comparing OMT with and without the addition of CPAP showed a significant difference (MD: -12.63; 95%CI: -17.62 to -7.63; I ^2^ : 0%; Fig. 3 ), while the subgroup analysis comparing OMT + CPAP versus CPAP alone showed no significant difference (MD: -0.48; 95%CI: -1.99 to 1.04; I ^2^ : 0%; Fig. 3 ); however, a significant difference was observed between these two subgroups ( *p* < 0.01).

**Fig. 4:**
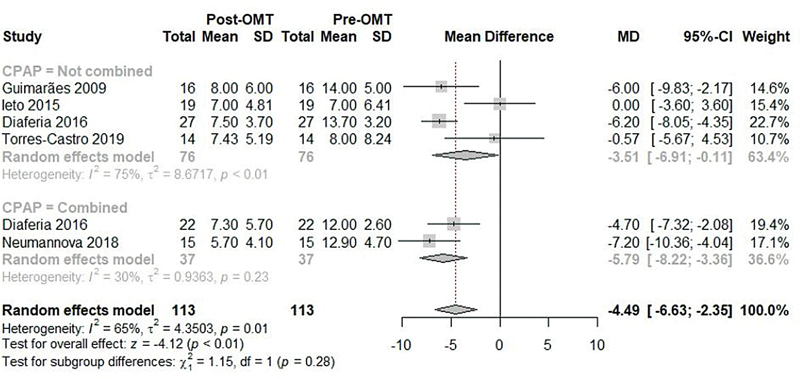
Meta-analysis of the ESS scores before and after OMT.

The meta-analysis of the mean ESS scores before and after OMT included the 5 studies 6 23 24 25 26 and showed a significant reduction after OMT, although with high heterogeneity (MD: -4.49; 95%CI: -6.63 to -2.35; I ^2^ : 65%; Fig. 4 ). The subgroup without association with CPAP showed a significant reduction, but with high heterogeneity (MD: -3.51; 95%CI: -6.91 to -0.11; I ^2^ : 75%; Fig. 4 ), while the subgroup with associated CPAP showed a more expressive reduction, with moderate heterogeneity (MD: -5.79; 95%CI: -8.22 to -3.36; I ^2^ : 30%; Fig. 4 ), with no significant difference between subgroups ( *p* = 0.28).

**Fig. 5:**
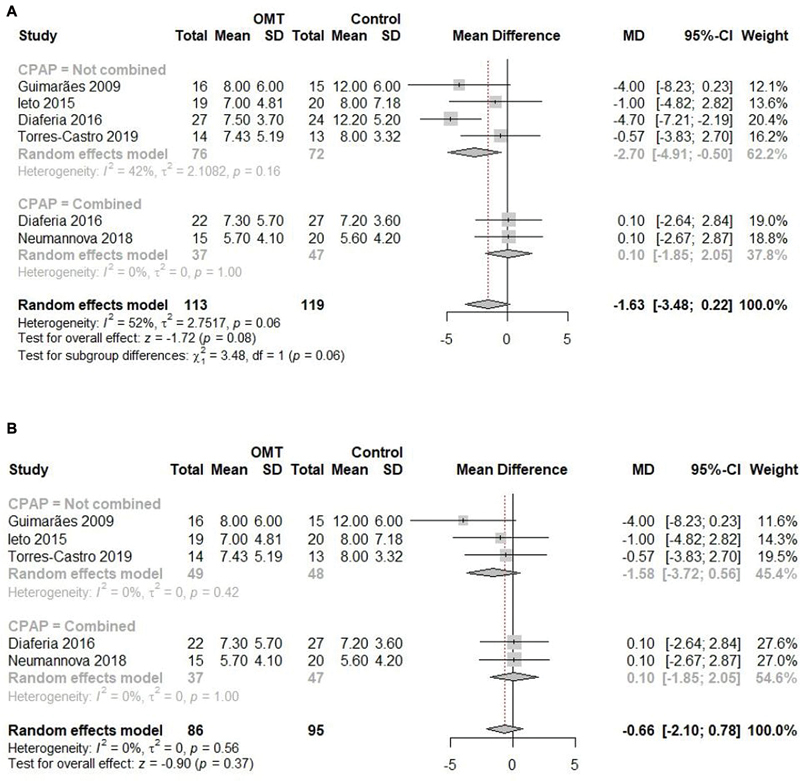
( **A** ) Meta-analysis of the ESS scores in the comparison of the OMT and control groups. ( **B** ) Meta-analysis of the ESS scores in the comparison of the OMT and control groups ater the sensitivity analysis.

In comparison with the control group, there was a non-significant difference regarding the mean ESS scores, with high heterogeneity (MD: -1.63; 95%CI: -3.48 to 0.22; I ^2^ : 52%; Fig. 5A ). The subgroup analysis comparing OMT with a control group without association with CPAP showed a significant difference, with moderate heterogeneity (MD: -2.70; 95%CI: -4.91 to 0.50; I ^2^ : 42%; Fig. 5A ), while OMT associated with CPAP did not present a significant difference compared with CPAP alone (MD: 0.10; 95%CI: -1.85 to 2.05; I ^2^ : 0%; Fig. 5A ), with no significant difference between subgroups ( *p* = 0.06).

In the sensitivity analysis, the removal of the OMT and control groups in the study by Diaféria et al. 25 (2017) explained all the statistical heterogeneity, with no significant difference among the groups in the global analysis (MD: -0.66; 95%CI: -2.10 to 0.78; I ^2^ : 0%; Fig. 5B ), neither regarding the subgroups without association with CPAP (MD: -1.58; 95%CI: -3.72 to 0.56; I ^2^ : 0%; Fig. 5B ) nor regarding those with association with CPAP (MD: 0.10; 95%CI: -1.85 to 2.05; I ^2^ : 0%; Fig. 5B ), with no significant difference between these subgroups ( *p* = 0.37).

**Fig. 6:**
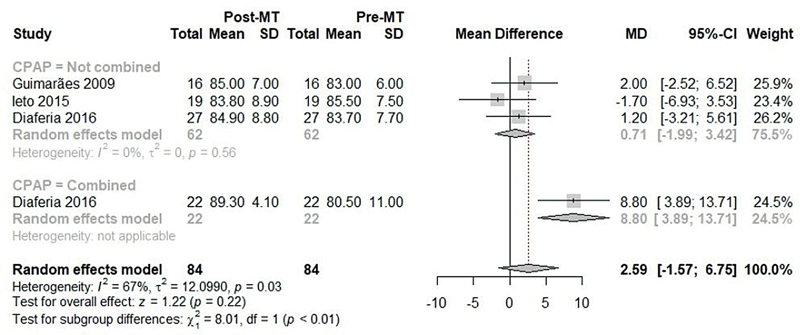
Meta-analysis of the SpO _2_ before and after OMT.

**Fig. 7:**
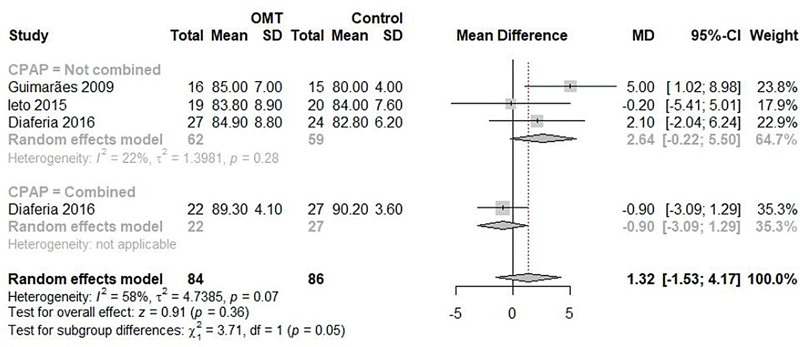
Meta-analysis of the SpO _2_ in the comparison of the OMT and control groups.

For the difference between means of the lowest SpO _2_ indices during PSG before and after OMT, 3 studies 23 24 25 were included, with no significant difference, with high heterogeneity (MD: 2.59; 95%CI: -1.57 to 6.75; I ^2^ : 67%; Fig. 6 ). Considering the subgroup with OMT without CPAP, there was no significant improvement in SpO _2_ (MD: 0.71; 95%CI: -1.99 to 3.42; I ^2^ : 0%; Fig. 6 ), but the effect of OMT associated with CPAP was reported by one study 25 as significant (MD: 8.80; 95%CI: 3.89 to 13.71; I ^2^ : not available; Fig. 6 ), with significant difference between subgroups ( *p* < 0.01). When comparing groups, no significant difference was identified either (MD: 1.32; 95%CI: -1.53 to 4.17; I ^2^ : 58%; Fig. 7 ), both in the subgroup without association with CPAP (MD: 2.64; 95%CI: -0.22 to 5.50; I ^2^ : 22%; Fig. 7 ) and in the study that compared OMT associated with CPAP and the isolated use of CPAP (MD: -0.90; 95%CI: -3.09 to 1.29; I ^2^ : not available; Fig. 7 ), with significant difference between subgroups ( *p* = 0.054).

**Fig. 8:**
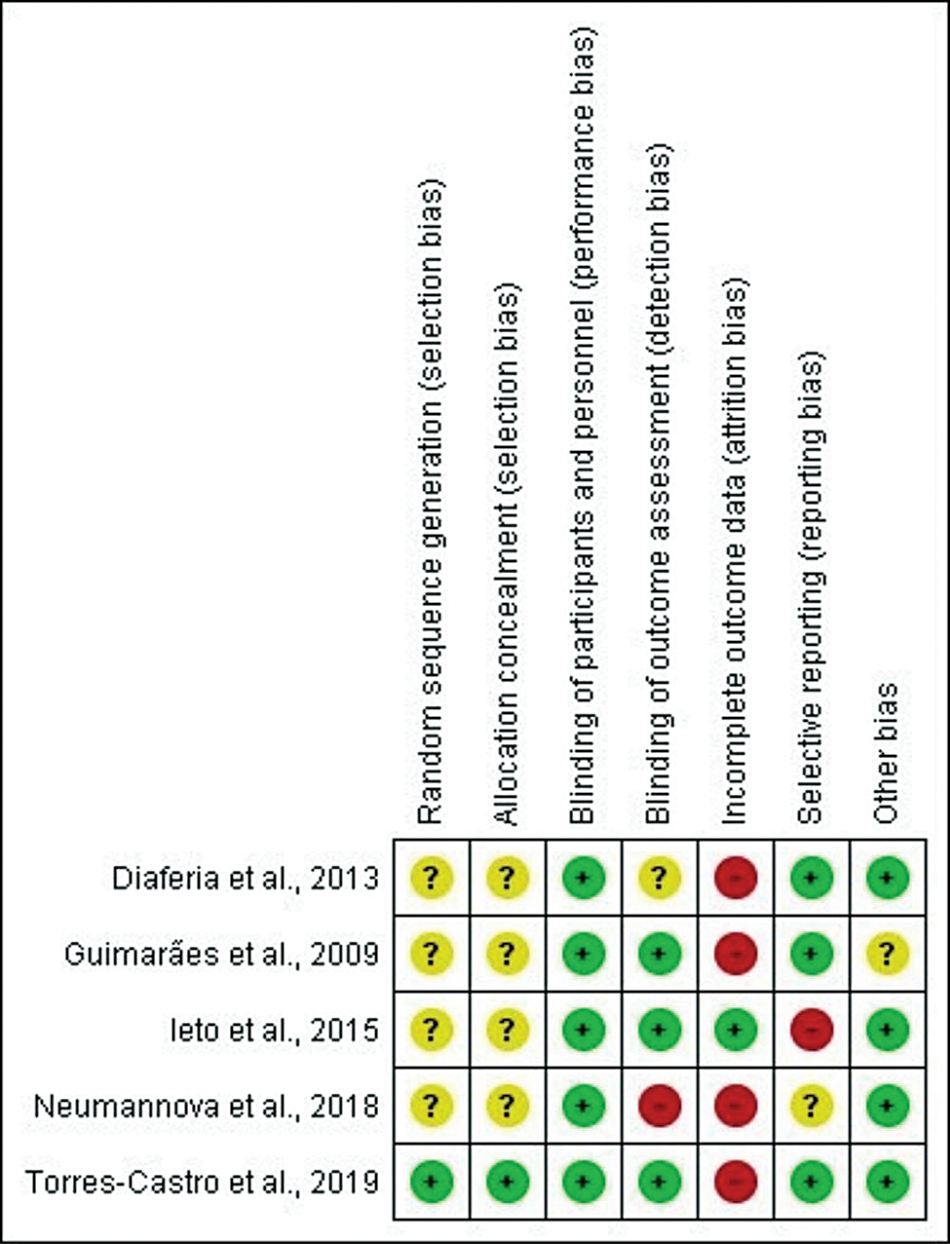
Analysis of the risk of bias.

The five articles 6 23 24 25 26 were evaluated using the Cochrane Collaboration's tool for assessing risk of bias tool 20 , as shown in Fig. 8 . When considering the item *not applicable* , in which the articles were classified when their methodology was not defined, but also did not point out biases, all articles presented a low risk of bias. The article by Torres-Castro et al. 6 was the only that presented all items classified as low risk of bias. The articles by Guimarães et al., 23 Ieto et al., 24 Diaféria et al., 25 and Neumannova et al. 26 had good ratings.

## Discussion

The present systematic review and meta-analysis of randomized clinical trials on the effect of OMT (alone or in combination with CPAP) in the treatment of OSA points out that OMT protocols lead to a reduction in the AHI (primary outcome) after 6 to 12 weeks of treatment, being superior to the control group, but not when compared with the use of CPAP. Regarding the other outcomes, a significant reduction in the ESS score was observed after OMT in study groups, but there was no significant difference in the comparison with the controls.

As aforementioned, the gold standard for the treatment of OSA is CPAP; however, the present systematic review aimed to list only the studies that used OMT to rehabilitate OSA either alone or in combination with the gold standard. Therefore, Neumannova et al. 26 (2018) and Diaféria et al. 25 (2017) submitted at least one group in their studies to an intervention that combined OMT and CPAP, which resulted in a significant reduction ( *p* < 0.01) in the AHI, demonstrating that, when performed together, the therapeutic results are enhanced. Still, when comparing OMT + CPAP with the CPAP control group, a significant difference was observed, demonstrating that, when the combined therapy is performed, better results are found in comparison with the isolated use of the gold standard. As observed by Diaféria et al., 25 the combination of OMT improves the adherence to CPAP use. A possible explanation would be that the combined groups were monitored more frequently than the CPAP group, which would justify the increase in adherence. Moreover, it is important to highlight the lack of studies that relate the effect of surgical procedures with therapy, which evidences that this relationship needs to be further explored to reach a conclusion about the final benefits to the patient.

In the analysis of the ESS score, there was a significant reduction in the indices with OMT, associated or not with CPAP, but with high heterogeneity. In the analysis of the subgroups, when comparing the subgroup submitted to isolated OMT and the control group, the removal of the study by Diaféria et al. 25 explained all the heterogeneity found, possibly due to the significant number of participants in this study in relation to the others; however, the statistical non-significance of the comparison of OMT versus control groups with or without associated CPAP for the ESS score was maintained.

As for the lower SPO _2_ , the study by Neumannova et al. 26 was not included in the specific meta-analysis due to the absence of minimum values for SPO _2_ , for the authors only reported the mean in their study. In the analysis of the remaining articles, there was no significant increase after OMT, neither was there a significant difference in the comparison with the controls. However, after the analysis of subgroups, a significant difference was observed in the OMT + CPAP group, indicating that the OMT will only have a significant result in the minimum SPO _2_ when accompanied by CPAP.

As limitations of the present study, the variability in the treatment protocols proposed in the included studies is highlighted, both in terms of duration and frequency of exercises, which can significantly affect the results estimated during the assessment of the risk of bias. We also emphasize that other relevant outcomes for the population with OSA, such as desaturation index, snoring intensity, and neck circumference, which may be affected by OMT, were not considered in the present review, and they deserve further investigation. Therefore, we consider that more randomized clinical trials, with high methodological rigor, should be performed to confirm the results estimated in the present systematic review.

Despite these limitations, we emphasize that the present study was conducted following the best practices recommendations in a systematic review, and is part of a collaboration with evidence-based practice in speech therapy.

## Final Comments

Based on the analyzed studies, we could verify the effectiveness of OMT in the treatment of adult OSA patients, both alone and in association with other interventions, through the reductions in the AHI and the ESS score. The OMT resulted in the highest mean of the lowest SpO _2_ index when associated with CPAP, with no effects verified with the isolated use of OMT. Even demonstrating efficacy, more studies with this focus are necessary, with less risk of bias and larger samples, to increasingly support the incorporation of OMT in the clinical practice directed at patients with OSA.
